# Transhumeral Arm Reaching Motion Prediction through Deep Reinforcement Learning-Based Synthetic Motion Cloning

**DOI:** 10.3390/biomimetics8040367

**Published:** 2023-08-15

**Authors:** Muhammad Hannan Ahmed, Kyo Kutsuzawa, Mitsuhiro Hayashibe

**Affiliations:** Department of Robotics, Graduate School of Engineering, Tohoku University, Sendai 980-8579, Japan; kutsuzawa@tohoku.ac.jp (K.K.); hayashibe@tohoku.ac.jp (M.H.)

**Keywords:** motion prediction, deep reinforcement learning, motion cloning, prosthetic elbow, artificial neural network

## Abstract

The lack of intuitive controllability remains a primary challenge in enabling transhumeral amputees to control a prosthesis for arm reaching with residual limb kinematics. Recent advancements in prosthetic arm control have focused on leveraging the predictive capabilities of artificial neural networks (ANNs) to automate elbow joint motion and wrist pronation–supination during target reaching tasks. However, large quantities of human motion data collected from different subjects for various activities of daily living (ADL) tasks are required to train these ANNs. For example, the reaching motion can be altered when the height of the desk is changed; however, it is cumbersome to conduct human experiments for all conditions. This paper proposes a framework for cloning motion datasets using deep reinforcement learning (DRL) to cater to training data requirements. DRL algorithms have been demonstrated to create human-like synergistic motion in humanoid agents to handle redundancy and optimize movements. In our study, we collected real motion data from six individuals performing multi-directional arm reaching tasks in the horizontal plane. We generated synthetic motion data that mimicked similar arm reaching tasks by utilizing a physics simulation and DRL-based arm manipulation. We then trained a CNN-LSTM network with different configurations of training motion data, including DRL, real, and hybrid datasets, to test the efficacy of the cloned motion data. The results of our evaluation showcase the effectiveness of the cloned motion data in training the ANN to predict natural elbow motion accurately across multiple subjects. Furthermore, motion data augmentation through combining real and cloned motion datasets has demonstrated the enhanced robustness of the ANN by supplementing and diversifying the limited training data. These findings have significant implications for creating synthetic dataset resources for various arm movements and fostering strategies for automatized prosthetic elbow motion.

## 1. Introduction

In recent decades, significant progress has been made in the development of advanced prosthetics [1,2] aimed at restoring lost limb function with multiple active degrees of freedom (DOF). However, despite the improvements in robotics and sensor technologies, there is a growing gap between control methods and hardware improvements, resulting in a rejection rate as high as 40% [3]. This disparity becomes even more pronounced in the case of transhumeral amputees.

The lack of intuitive controllability remains a primary challenge in enabling transhumeral amputees to control a multi-functional prosthesis, which includes a powered hand, wrist, and elbow, replicating various functions of a human arm. A significant control objective is the execution of elbow joint motion and wrist pronation–supination during target reaching tasks. Currently, commercially available prosthetic elbows are controlled through electromyographic (EMG) signals, which results in complex control schemes and the development of compensatory strategies involving large trunk and shoulder displacements [4]. However, the myoelectric control strategy lacks intuitiveness since the physiologically appropriate muscles are unavailable, necessitating highly invasive surgeries such as targeted muscle reinnervation (TMR) to overcome these limitations [5]. Furthermore, an analysis of the manipulation strategies employed by prosthetic users [6] suggests that body-powered devices tend to offer more intuitive control compared to myoelectric devices. It has been observed that myoelectric devices often make routine tasks more cumbersome and time-consuming to perform [7]. As an alternative, bio-inspired and human motor-control-based techniques have been developed to achieve more natural control of multiple DOFs [8,9].

Recent studies have focused on enhancing the intuitive control of prosthetic elbow joints by leveraging movement synergies that govern coordinated joint movements in the upper limb [10,11,12]. Previous studies have revealed that human movements can be effectively characterized by a reduced set of primitive components known as motor synergies [13,14,15]. It has also been observed that similar movements performed by different individuals exhibit shared synergies, indicating the reusability of motor synergy patterns [16,17]. Researchers in [18] have successfully demonstrated the generalization ability of movement synergies for new targets in multi-directional scenarios.

Building upon this concept, recent studies such as [19,20] have showcased the effectiveness of controlling wrist pronation–supination and elbow flexion–extension through remaining shoulder movements, which participants have found intuitive. This approach combines the residual limb motion strategy with the predictive capabilities of ANNs, harnessing the inherent movement synergies between the shoulder and elbow joints. As a result, it enables independent and simultaneous control of the multi-DOF prosthesis. In particular, radial basis function network (RBFN) models have proven effective in capturing intricate inter-joint coordination patterns in various ADLs [21,22]. Additionally, ANNs and fuzzy logic methodologies have been successfully applied for classifying and predicting prosthetic arm motions [23]. Moreover, the combination of EMG and shoulder orientation data has also been explored to estimate distal arm joint angles [24].

However, one of the crucial challenges associated with this strategy revolves around the acquisition of a sufficient amount of training data from human experiments, as this approach relies on ANNs to identify and model the intricate coordination between the shoulder and elbow joints. This necessitates providing extensive training data to the network during the learning process. Obtaining such training data involves expensive motion capture equipment and a lengthy, repetitive process where subjects perform numerous repetitions of the desired ADLs. The quantity and quality of the motion data obtained also significantly impact the performance of the ANN, as there are certain levels of motion variations among different human subjects.

This study presents an innovative motion-cloning strategy to address the challenge of acquiring a substantial amount of training data for effective training of ANN. Our approach leverages the capabilities of DRL algorithms to create natural and human-like motion in simulated humanoid agents [25]. We introduce a DRL-based motion cloning framework that utilizes a 7-DOF robot arm model in a mujoco simulation to generate synthetic motion data. Furthermore, we explored the use of the synthetic motion data obtained from DRL simulation (hereafter referred to as DRL-Data) to train different ANNs and demonstrate the effectiveness of DRL-Data in accurately estimating the arm motion of human subjects by comparing it with their actual motion data (hereafter referred to as Real-Data). Moreover, the integration of Real-Data and DRL-Data through motion data augmentation demonstrated the enhanced robustness of the trained ANNs. This approach addresses the challenge of limited motion data availability by supplementing and diversifying the training data, thereby improving the ANN’s ability to generalize across different subjects.

The fundamental concept behind the proposed framework is that the simulated robot arm has the ability to learn and replicate a wide range of desired movements. We can utilize the extracted motion data from the shoulder and elbow joints from the simulated arm to effectively supplement and diversify the training data for the ANN. To the best of our knowledge, our study represents the first successful demonstration of employing learning–synthetic motion data to estimate actual human arm movements.

This paper is organized as follows. Section 2 presents our proposed framework, including details of the experimental protocols and the implementation of DRL simulation. The ANN training strategy and the method employed for performance evaluation are also described in this section. The results are presented in Section 3 and discussed in Section 4. Finally, we draw conclusions and discuss future works in Section 5.

## 2. Materials and Methods

### 2.1. Experiment Protocols

**Figure 1:**
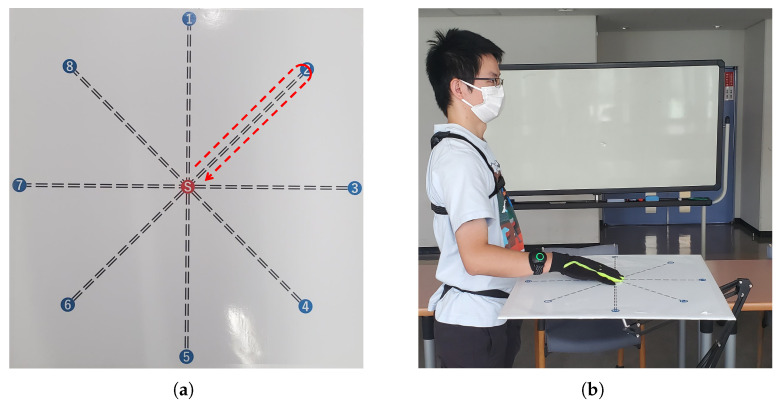
The designed experimental protocol for arm reaching movements in the horizontal plane: (**a**) The target points are arranged in a circular pattern. The center point (red) represents the initial neutral/rest position, and the outer points (blue) numbered 1 to 8 indicate the target points to be reached. The arrow depicts the outline of the desired center-out-center reaching movement to be performed. (**b**) An illustration of a subject with the target grid in the horizontal plane, demonstrating the positions of the target points relative to the participant.

This study focuses on estimating the elbow joint motion and wrist pronation–supination during arm reaching movements, spanning across multiple directions in the horizontal planes. We designed our experiment by drawing inspiration from the investigation carried out in [26], which explored arm reaching movements towards target points arranged in a circular manner. We created a target grid consisting of eight points positioned along the circumference of a circle with a diameter of 0.5 m, as depicted in Figure 1a.

The experimental task involves a reaching movement starting from a resting position at the center point, then reaching and touching the selected outer target point, and finally returning to the center point. A brief pause at the center point precedes the repetition of the process to reach the next target. This movement task is referred to as the center-out-center reaching task. Throughout the experiments, the participants were instructed to perform center-out-center reaching movements towards all eight target points within the horizontal plane, as illustrated in Figure 1b.

### 2.2. Human Subject Motion Data Acquisition: Real-Data

Six right-handed individuals (five males and one female) with no known upper limb neuromuscular disorders volunteered as participants for this study. The age range of the subjects was between 20 and 28 years old. Prior to the experiment, all participants provided informed consent to be involved in the research.

**Figure 2:**
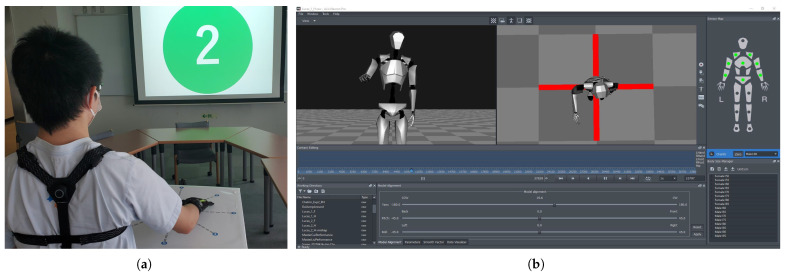
Experimental setup for capturing arm reaching motion data from human subjects. (**a**) A human subject wearing the neuron pro motion capture system and performing the reaching motion on the target grid in the horizontal plane, with the desired target point projected on the front screen. (**b**) Illustration of axis neuron pro software with a real-time 3D model.

To acquire arm motion data from the participants, we implemented an experimental setup as depicted in Figure 2. The participants were instructed to execute center-out-center reaching movements with their right arm while standing. A number was displayed on a screen in front of the subjects, indicating the specific target point to be reached (Figure 2a). The timing of the movements was controlled passively through automatic color changes at fixed intervals. The green color indicated the start of the reaching movement towards the outer target point, and it remained green for 2 seconds. A display of red color indicated the return to the center point and the waiting phase, as it remained red for 5 seconds. The process was repeated for the next movement once the color turned green again.

We employed neuron pro, an inertial measurement unit (IMU)-sensor-based full-body motion capture system, to capture the participants’ motion data. While this system’s accuracy may be lower than that of optical-camera-based motion capture systems, it offers the advantage of capturing motions without spatial constraints from any location within the device’s communicable range. The neuron pro system includes the axis neuron pro software (Figure 2b), which processes raw IMU data and generates a real-time 3D skeletal model. This skeletal model provides valuable motion information, including the position and angle of each joint, which can be saved for further analysis.

The participants were instructed to perform only four repetitions of the center-out-center reaching movements for each target point, resulting in a relatively small amount of collected data. We conducted the experiment twice per subject, recording two sets of motion data, one each for training and testing purposes. The motion data of interest included the angles of the right arm’s 3-DOF shoulder joint (rotation ($S\theta_{x}$), flexion ($S\theta_{y}$)), and abduction ($S\theta_{z}$) and 2-DOF elbow joint (pronation ($E\theta_{x}$) and flexion ($E\theta_{y}$)) during the reaching movements toward the targets. These joint angles were saved and utilized for cross-validation testing of the trained ANNs to assess the effectiveness of the DRL-Data approach.

### 2.3. Deep Reinforcement Learning (DRL)-Based Motion Cloning: DRL-Data

The model-based approach, which involves mathematical optimization for addressing high-dimensional or redundancy problems in robotics, requires prior knowledge of robot dynamics and the operating environment. In contrast, DRL presents a promising model-free strategy that learns an effective policy through iterative trial-and-error interactions with the environment without relying on dynamic parameters such as mass, inertia, or even the model itself. An essential aspect is designing a suitable reward function, as DRL algorithms enable robotic agents to learn optimal actions by maximizing cumulative rewards within their virtual environment. In a related study [27], quantitative evidence was provided to demonstrate that deep learning, like humans, also exhibits motor synergy, enabling robotic agents to achieve energetically efficient and natural human-like motion.

**Figure 3:**
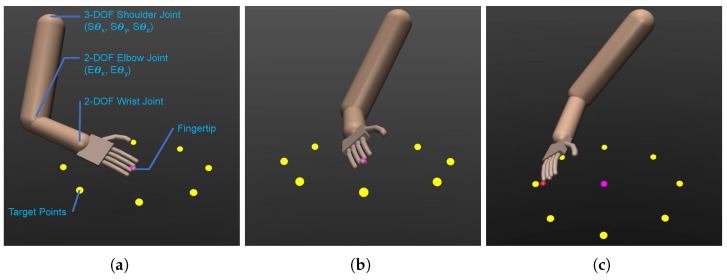
The simulated anthropomorphic 7-DOF robot arm and the target points in the MuJoCo simulation environment showcasing the setup: (**a**) Isometric view of the simulated robot arm with the joints and a description of the DOFs. (**b**) The simulated robot arm in a neutral pose, with target points arranged horizontally. (**c**) The simulated robot arm tracking a moving point (red) to reach and touch a designated target point (yellow).

To generate synthetic motion data according to the predefined experimental protocols, we utilized MuJoCo [28], a widely used simulation engine in the DRL research community for studying multi-joint mechanical systems. We created an anthropomorphic 7-DOF robotic arm agent consisting of three sequentially connected modules: a 3-DOF shoulder joint with abduction, flexion, and rotation, a 2-DOF elbow joint with flexion and pronation, and a 2-DOF wrist joint with abduction and flexion, mimicking a human arm’s configuration. This arrangement, depicted in Figure 3a, replicates the total DOFs of a real human arm, considering the forearm’s axial rotation as part of the elbow joint articulation along with elbow bending. The arm’s endpoint is positioned at the fingertip of the middle finger.

Our study utilizes the advanced soft actor–critic (SAC) algorithm [29] for synthetic motion generation. In the DRL domain, tasks are typically represented as infinite-horizon Markov decision processes (MDP), characterized by the tuple (𝒮,𝒜,p,r). Here, 𝒮 denotes the continuous state space, 𝒜 represents the possible action space, and p:𝒮×𝒜×𝒮→[0,∞) defines the probability density of transitioning from the current state $s_{t}\in 𝒮$ to the next state $s_{t+1}\in 𝒮$ given the action $a_{t}\in 𝒜$. Additionally, r:𝒮×𝒜→R is the reward function, providing a scalar reward at each transition. The trajectory distribution induced by a policy $\pi (a_{t}|s_{t})$ is denoted by $\rho_{\pi }$. The SAC algorithm is a cutting-edge stochastic DRL technique that learns a policy $\pi (a_{t}|s_{t})$ aiming to maximize not only the rewards but also the expected entropy $E_{\rho_{\pi }}\left[H\left(\pi \left(\cdot |s_{t}\right)\right)\right]$, weighted by an entropy term α as expressed in Equation (1). This maximization of expected entropy enhances the exploration of diverse behaviors during training, accelerating learning and significantly reducing sub-optimal solutions.

(1) $$\pi_{SAC}=E_{\left(s_{t},a_{t}\right)∼\rho_{\pi }}\left[r\left(s_{t},a_{t}\right)+\alpha \cdot H\left(\pi \left(\cdot |s_{t}\right)\right)\right]$$

To make the 7-DOF robot arm learn the reaching motion toward the target points, it is assigned a task to track and follow a moving point. The trajectory of the moving point adheres to the prescribed center-out-center reaching task outlined in the experimental protocols. Starting at the center point, the moving point commences a linear movement towards a designated target in yellow (Figure 3b), aiming to reach and touch it, followed by a return to the center point. A brief pause occurs at the center point before the moving point transitions to the next target, ensuring that all eight target points are periodically reached during the training phase. Utilizing the SAC algorithm, the policy is learned to enable the robot arm’s endpoint (the fingertip of the middle finger) to accurately track the moving target (as depicted in Figure 3c). The objective is to minimize the position error between the endpoint and the moving target in the task space while ensuring energy-efficient motion.

The reward function used during training is defined as in Equation (2) and has three terms with constant coefficients *b*, *c*, and *d* carefully chosen through experimentation to maximize performance potential. The position error $error_{p}$ quantifies the distance between the moving point’s position and the current fingertip position computed from the state. This component enables the robot arm to effectively learn the center-out-center reaching motion. The ∥.∥ notation represents the Euclidean Norm and captures the total energy cost associated with each action $a_{t}$. By minimizing this term, the robot arm is encouraged to optimize energy usage, resulting in the generation of synergistic motion that closely resembles human-like behavior. Furthermore, the palm orientation error “$error_{o}$” ensures proper palm orientation during the reaching motion, aligning with the observations made during the human motion data acquisition experiments, where the palm of the hand consistently faced downwards.

(2) $$r\left(s_{t},a_{t}\right)=-b\cdot error_{p}-c\cdot {∥a_{t}∥}^{2}-d\cdot error_{o}$$

The training involved learning the reaching movements towards the target points in the horizontal plane over 200,000 steps. Upon completion, the robot arm’s learned motion was simulated within the MuJoCo environment, where it successfully executed the center-out-center reaching tasks for all target points. Subsequently, synthetic motion data were extracted from the robot arm during the target reaching movements, comprising 3-DOF shoulder joint angles (i.e., rotation, flexion, and abduction) and 2-DOF elbow joint angles (i.e., pronation and flexion). These joint angles are hereafter referred to as $S\theta_{x}$, $S\theta_{y}$, $S\theta_{z}$, $E\theta_{x}$ and $E\theta_{y}$, respectively, constituting our motion dataset. We acquired the DRL-Data containing four repetitions of reaching movements per target point, later used as the training dataset for the ANNs.

### 2.4. Convolutional Long Short-Term Memory (CNN-LSTM) Neural Network

This study employed convolutional long short-term memory (CNN-LSTM) neural networks to train a neural network model capable of recognizing shoulder–elbow coordination and predicting elbow joint angles based on shoulder kinematics input. The CNN-LSTM architecture combines the strengths of both convolutional neural networks (CNNs) and long short-term memory (LSTM) recurrent neural networks (RNNs), which have shown promising results in various time series prediction or classification tasks. Recent studies, such as human activity recognition in [30], have explored combining CNN and LSTM layers to enhance performance. This combination is motivated by the idea that LSTM’s performance can be limited by the quality of the input features it receives [31]. By incorporating CNN layers, which are adept at reducing input frequency variance and extracting meaningful features, we improve the overall feature representation. The LSTM layers then capture temporal dependencies within the extracted features. Additionally, our approach integrates CNN and LSTM layers within a unified architecture, allowing optimized training for all layers.

We utilized Python’s machine learning library, Keras, to implement our CNN-LSTM model. Following the methodology outlined in [22], our CNN-LSTM network was trained to establish the relationship between shoulder and elbow joint angles during target reaching movements. The training process involved supervised learning, where the CNN-LSTM developed a regression model using input–output pairs. Specifically, the CNN-LSTM received the shoulder joint angles ($S\theta_{x}$, $S\theta_{y}$ and $S\theta_{z}$) as input and was trained to predict the corresponding elbow joint angles ($E\theta_{x}$ and $E\theta_{y}$).

**Figure 4:**
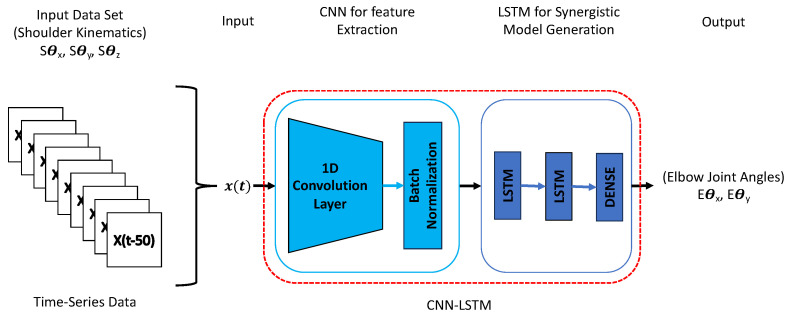
CNN-LSTM model architecture.

To achieve high prediction accuracy, we fine-tuned the hyperparameters of the CNN-LSTM network, including the number of nodes and hidden layers. We found that a CNN-LSTM network with a single one-dimensional CNN layer, two LSTM layers, each containing 256 nodes, and a final dense layer along with the Adam optimization function yielded efficient results (Figure 4). This configuration allowed the network to effectively capture features from the input data and model the temporal dependencies necessary for accurate estimation.

### 2.5. Analysis Strategy

To assess the effectiveness of the DRL-Data, we trained various CNN-LSTM models using different input data configurations. In particular, we tested two different scenarios:

#### 2.5.1. Sufficient Human Motion Data Availability

In the first scenario, we evaluated how well the DRL-Data could be used for predicting the elbow joint motion of a human arm during reaching movements across different subjects. For performance comparison, we developed two CNN-LSTM models: the *DRL-Model* and the *Human-Avg-Model*. The *Human-Avg-Model* was trained using an averaged motion dataset from five human subjects, making it suitable for the sufficient data scenario. On the other hand, the *DRL-Model* was solely trained on synthetic motion data. We then evaluated the performance of both models using a new motion dataset from a sixth subject. Below are the details of the trained CNN-LSTM models:

- *DRL-Model:*
    *DRL-Model* was trained with synthetic motion data generated from a DRL simulation without using any human joint angle information. Its purpose was to evaluate how well the decoder based only on DRL-Data could predict elbow joint motion in human arms during reaching movements across different subjects.
- *Human-Avg-Model:*
    Six distinct *Human-Avg-Models* were trained using averaged motion data obtained by combining the motion data from five human subjects. Each *Human-Avg-Model* excluded the motion data from one of the six subjects, which was later utilized for testing the model. These *Human-Avg-Models* served as the gold standard for performance comparison given that the predictive model trained using motion data from multiple human subjects’ can capture typical features of human reaching motion from Real-Data.
- *Performance Assessment:*
    The efficacy of the trained CNN-LSTM models (*DRL-Model* and *Human-Avg-Model*) was tested using real motion data captured via the neuron pro motion capture system. For example, to validate the *Human-Avg-Model*, which was trained on averaged motion data from subjects two to six (S2–S6), subject one’s (S1) motion data were employed. Subsequently, subject one’s (S1) motion data were also used to test the *DRL-Model*. For comprehensive performance evaluation, the predicted elbow joint motion angles from both CNN-LSTM models were compared with the original elbow joint motion data of subject one (S1) and quantified using the root mean squared error (RMSE) (Section 2.6.2), a key metric for performance assessment. This iterative testing procedure was replicated using the motion data of each subject to ensure thorough performance evaluation across multiple subjects.

#### 2.5.2. Limited Human Motion Data Availability

In the second scenario, we focused on exploring the potential enhancement of performance and efficiency in a CNN-LSTM model through motion data augmentation by the integration of DRL-Data with Real-Data. For comparison purposes, we trained two types of CNN-LSTM models: the *Hybrid-Model* and the *Human-Sparse-Model*. This scenario is considered a limited data scenario as we utilized motion data from a single human subject with a constraint. To elaborate, for training the *Human-Sparse-Model*, the motion data comprised one repetition of reaching movements towards only four out of the eight target points (specifically, target points 1, 3, 5, and 7).

Conversely, the *Hybrid-Model* was trained using an augmented motion dataset. We combined the motion data from the same human subject as before and enriched it with DRL-Data consisting of reaching movements towards an additional set of four target points (target points 2, 4, 6, and 8), thereby diversifying the training dataset.

To assess the performance of both CNN-LSTM models comprehensively, we evaluated their ability to predict reaching motions towards all eight target points utilizing new motion data from five different subjects, employing a cross-subject evaluation strategy. Details of the trained CNN-LSTM models are as follows:

- *Hybrid-Model:*
    Six distinct *Hybrid-Model*s were trained using the augmented motion dataset, which combined the motion data from only one human subject, having one repetition of reaching movements toward four target points, and the DRL-Data, with one repetition of reaching movements toward four additional target points (eight target points in total). The aim was to investigate the potential of the DRL-Data to supplement and diversify the limited training data, thereby enhancing the performance of the CNN-LSTM model.
- *Human-Sparse-Model:*
    Six separate *Human-Sparse-Model*s were trained, each using motion data from only one human subject, with the limitation of having only one repetition of reaching movements towards the specified four target points. This *Human-Sparse-Model* establishes a baseline for performance comparison and evaluation of the corresponding *Hybrid-Model*s.
- *Performance Assessment:*
    The effectiveness of the augmented motion data was assessed through a comparative analysis of the predictive capabilities of the *Hybrid-Model* and *Human-Sparse-Model*. This validation process was conducted using real motion data encompassing reaching movements toward all eight target points, employing a cross-subject methodology. To illustrate, if subject one’s (S1) motion data were utilized to train the *Human-Sparse-Model* and augmented motion data from subject one (S1 + DRL) were employed for training the *Hybrid-Model*, then the performance of both models was evaluated using a cross-subject approach, involving motion data from subjects two to six (S2–S6). The root mean squared error (RMSE) (as described in Section 2.6.2) between the predicted and original elbow joint angular values was computed, serving as a performance assessment metric.

### 2.6. Evaluation

To evaluate the quality of the synthetic motion data and assess the performance of the CNN-LSTM models in predicting elbow joint angles, we employed well-established metrics such as Pearson’s correlation coefficient and root mean squared error (RMSE) [32]. By utilizing Pearson’s correlation coefficient, we could assess the degree of linearity between the predicted joint angles and the actual values, providing insights into the model’s ability to capture the underlying synergistic patterns in the data, whereas the RMSE metric enables us to gauge the overall accuracy and precision of the CNN-LSTM models’ estimations.

#### 2.6.1. Pearson Correlation Coefficient

Pearson’s correlation method examines the linear relationship between two variables and quantifies the strength of their correlation. The resulting coefficient, denoted as *“r”*, ranges between −1 and +1, offering insights into the extent and direction of the correlation. Table 1 presents the detailed interpretation of the Pearson correlation coefficient.

To compute the Pearson correlation coefficient, we employed the “corrcoef” function available in Python’s NumPy library, which uses the subject’s original elbow joint angles and the CNN-LSTM estimated elbow joint angles to compute the Pearson correlation coefficient.

#### 2.6.2. Root Mean Squared Error (RMSE)

For performance evaluation, we compared the estimated elbow joint angles (i.e., pronation–supination $E\theta_{x}$ and flexion–extension $E\theta_{y}$) with the subject’s original elbow joint angles obtained during the reaching movements captured using the neuron pro system, using the root mean squared error (RMSE) metric as defined in the Equation (3). Here, ${\hat{x}}_{t}$ is the predicted joint angle and $x_{t}$ is the actual joint angle at data point *t*. The total number of data points is represented by *N*.

(3) $$\mathrm{RMSE}=\sqrt{\frac{1}{N}\sum_{t=0}^{N}{\left({\hat{x}}_{t}-x_{t}\right)}^{2}}$$

#### 2.6.3. Target Point Reaching Error: Unity 3D Simulation

**Figure 5:**
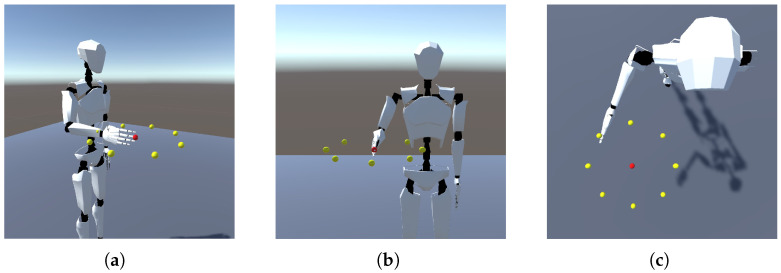
The Unity 3D simulated environment for estimated motion animation: (**a**) Isometric view showcasing the arrangement of target points horizontally in a circular configuration, with the humanoid actor positioned in a standing stance. (**b**) Front view of the humanoid actor in a neutral pose, with the middle finger’s fingertip at the center point. (**c**) Illustration of the humanoid actor’s arm reaching towards a designated target point (yellow).

To evaluate the accuracy of the predicted arm movements in reaching the target points, we utilized a Unity 3D simulation. This simulation was designed to animate the motion data predicted by the CNN-LSTM model. The Unity simulation replicated the setup of the human subject motion data acquisition experiment, featuring a humanoid actor in a standing position with target points arranged in the horizontal plane (as shown in Figure 5).

The joint angles from each subject’s original motion data and the corresponding predicted elbow joint angles from both the *DRL-Model* and the *Human-Avg-Model* were used for the animation. This allowed the humanoid actor to visualize the arm reaching movements toward all the target points. The target point reaching error of both the CNN-LSTM models was determined relative to the actual arm reaching movements of each human subject toward the specified target points animated in the Unity 3D simulation. The Unity-based evaluation provided valuable insights into the performance of the CNN-LSTM models through the visualization of the predicted arm reaching motions.

## 3. Results

In this section, we present the analysis results of our proposed DRL-based synthetic motion cloning approach. Firstly, we demonstrate that the generated DRL-Data accurately replicate synergistic human-like motion and exhibit joint angular movement patterns similar to those observed in human subjects during arm reaching motions. Next, we showcased the effectiveness of the *DRL-Model*, a CNN-LSTM model trained using the DRL-Data as input, in predicting the elbow joint motion of different human subjects. The *DRL-Model* achieves comparable performance to the gold-standard *Human-Avg-Model*. Most notably, our cross-subject evaluation reveals that motion data augmentation through the combination of Real-Data and DRL-Data can improve the performance of sparse CNN-LSTM models (*Hybrid-Model*) in scenarios with limited data availability.

**Figure 6:**
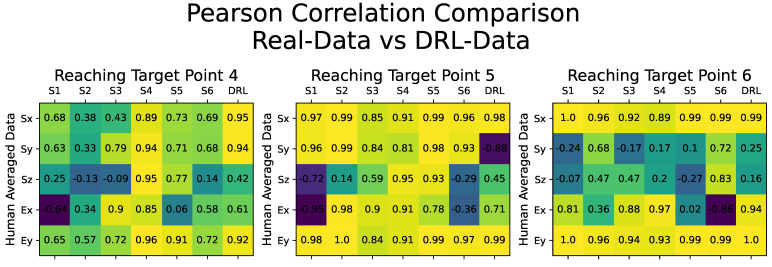
Confusion matrices illustrating the Pearson’s correlation coefficients obtained by comparing the motion datasets with the averaged human motion data from all six subjects. Each confusion matrix presents Pearson’s correlation comparison for reaching movements toward a specific target point. The 3-DOF shoulder and 2-DOF elbow joint angular values (Sx, Sy, Sz, Ex, and Ey) are compared. The correlation values are displayed within small boxes, with lighter colors (yellow) indicating stronger correlations and darker colors (green, purple, etc.) representing weaker correlations. The columns labeled S1 to S6 depict the comparison with motion data from each subject, while the last column (DRL) compares the generated synthetic motion data.

Starting with the quality assessment of the synthetic motion data, our analysis focused on evaluating the correlation between the DRL-Data and the averaged human motion dataset from all six subjects, which served as the benchmark for this comparison. Figure 6 illustrates the results through a confusion matrix, presenting the Pearson’s correlation coefficient obtained by comparing all of the motion datasets, including the Real-Data for each human subject and the DRL-Data.

Figure 6 comprises individual confusion matrices demonstrating Pearson’s correlation comparison for reaching movements towards target points, specifically the target points numbered 4, 5, and 6. The columns labeled S1 to S6 depict the comparison with motion data from each subject sequentially, while the final column presents the comparison with the generated DRL-Data. Variations in Pearson’s correlation values can be observed due to the subjects’ inherent individual differences in reaching motion. However, the overall trend highlights the consistency among all participants and the DRL-Data, indicating a shared movement pattern. The similarity between the Pearson’s coefficient values of the DRL-Data and those of any other human subject’s motion data suggests that the synthetic dataset generated by our DRL-based motion cloning framework can be considered as an additional subject within the experiment.

The next step involved evaluating our framework’s effectiveness in predicting elbow motion during actual human arm reaching movements. To achieve this, we utilized the synthetic motion data as the training dataset for a CNN-LSTM network called the *DRL-Model* (see Section 2.5). As the DRL-based motion cloning framework aimed to replicate human-like motion, we expected the performance of the *DRL-Model* to be comparable to that of the *Human-Avg-Model* (see Section 2.5), which was trained using the averaged human motion dataset. Although slight variations in performance were expected due to the artificially generated nature of the DRL-Data, we anticipated that it would capture the essence of reaching movement synergistic patterns to effectively train the *DRL-Model*. After training both models, we employed them to predict the elbow joint angles ($E\theta_{x}$ and $E\theta_{y}$) during reaching motions performed by actual human subjects. Their shoulder joint angles ($S\theta_{x}$, $S\theta_{y}$ and $S\theta_{z}$) served as input for the estimation process (see Section 2.4). Subsequently, the estimated elbow joint angular values were compared to the subjects’ original elbow joint angular values to analyze performance.

**Figure 7:**
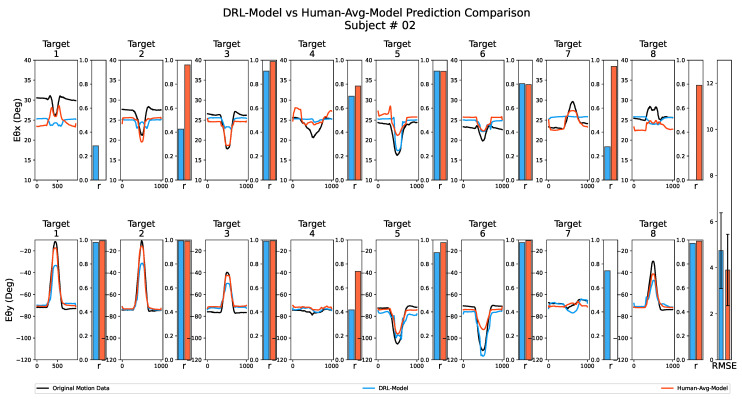
Prediction performance analysis for both the *DRL-Model* and the *Human-Avg-Model* for one of the subjects. The top row illustrates the elbow pronation–supination angle $E\theta_{x}$, while the bottom row represents the elbow flexion–extension angle $E\theta_{y}$. The line graph visually represents the joint angle variation during reaching movements toward each target point. The original joint angles are displayed in black, the *DRL-Model* estimations are shown in blue, and the *Human-Avg-Model* estimations are depicted in red. The adjacent bars correspond to Pearson’s correlation coefficient values for each comparison, while the overall RMSE value is depicted in the bar graph in the last column, with the error bar representing the standard deviation of estimation error values.

Figure 7 displays the results of the prediction performance analysis for both the *DRL-Model* and the *Human-Avg-Model* for one of the subjects. The line graph illustrates the variation in the joint angles (elbow pronation–supination $E\theta_{x}$ and flexion–extension angle $E\theta_{y}$) during reaching movements towards each target point, while the adjacent bars indicate the corresponding Pearson’s correlation coefficient values compared to the subject’s original joint angular variation. Additionally, as depicted in the bar graph in the last column, we computed the overall RMSE value by comparing the estimated and original joint angular values for all target points along with the error bar representing the standard deviation of the estimation error values. As suggested by similar Pearson’s correlation coefficient values and slight differences in overall RMSE values in Figure 7, both the *DRL-Model* and the *Human-Avg-Model* exhibited comparable performance.

**Figure 8:**
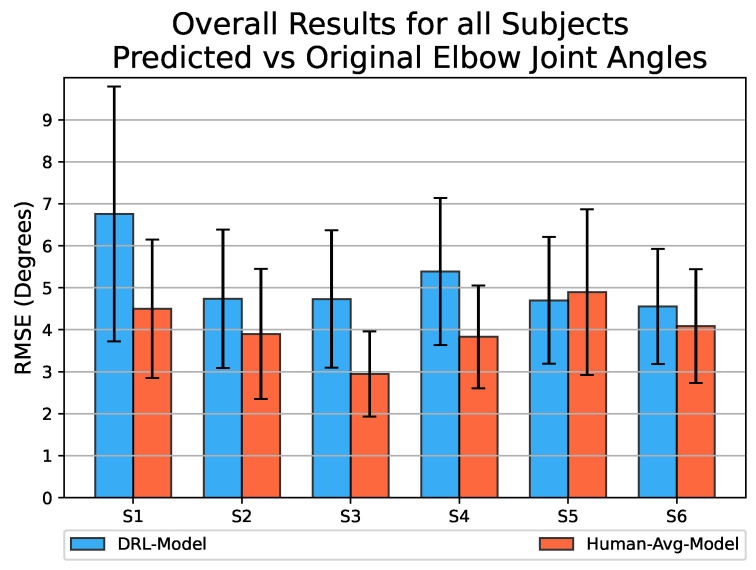
Bar graph representing the overall RMSE values obtained by comparing the estimated elbow joint angular values to the original values for reaching movements towards all target points, using both the *DRL-Model* (shown in blue) and the *Human-Avg-Model* (shown in red) across all participating subjects. The error bar represents the standard deviation of estimation error values.

The bar graph in Figure 8 presents the overall RMSE values obtained for the prediction of elbow joint motion across all six participating subjects. Since the *Human-Avg-Model* was trained using an averaged human motion dataset as input, it is expected to have good prediction results. Although the *DRL-Model* with an overall average RMSE value of 5.14° exhibits slightly lower performance compared to the *Human-Avg-Model* with an overall average RMSE value of 4.03°, the results demonstrate its successful prediction of elbow joint angles with sufficient accuracy for all subjects.

This highlights the effectiveness of synthetic motion data in training a neural network model for predicting natural human motion. Notably, for testing the *DRL-Model*, human motion data are used as input, which differs entirely from the synthetic motion dataset used for training. However, it still achieves reasonable accuracy in predicting elbow joint motion for all subjects. This highlights the robustness of the *DRL-Model* in accommodating inter-subject variability.

Furthermore, we analyzed the target point reaching error associated with the predicted elbow joint motion for both the *DRL-Model* and the *Human-Avg-Model*. For this evaluation, we utilized a Unity 3D simulation with a humanoid actor, as detailed in Section 2.6.3. This animation process involved visualizing not only the predicted motion data generated by both the *DRL-Model* and the *Human-Avg-Model* but also the subject’s original motion data. During the simulation, we tracked the position of the middle finger’s fingertip as the humanoid actor executed the reaching movements toward each designated target point. The target point reaching error was determined relative to the actual arm reaching movements for each individual subject, as animated in the Unity 3D simulation.

**Figure 9:**
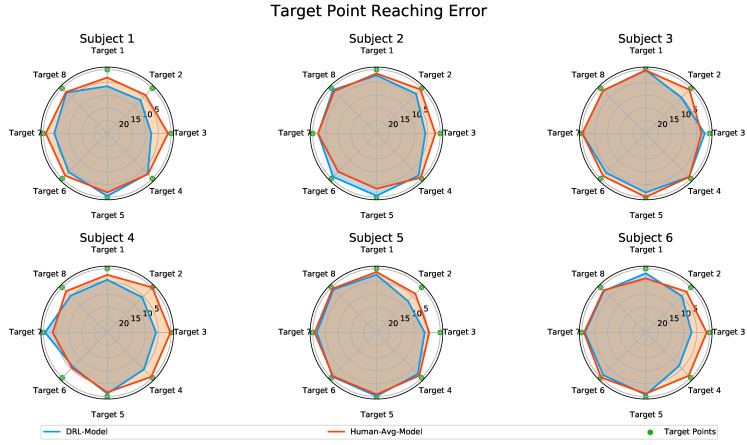
Comparison of the target reaching error for motion predictions of the *DRL-Model* and the *Human-Avg-Model*. The polar charts present the target reaching error for each point across all subjects for the *DRL-Model* in blue and the *Human-Avg-Model* in red, where the radial axis indicates the scale of the position error measured in centimeters.

Figure 9 presents a comparison of the target reaching error based on the predictions made by the *DRL-Model* and the *Human-Avg-Model*, illustrated on polar charts. The predicted motion data from both the *DRL-Model* and the *Human-Avg-Model* demonstrated the successful reaching of most target points with reasonable accuracy across all subjects. Although there were slight variations in a few cases, the overall trend of final position errors was similar for both models, with an average overall mean value of 3.03 cm for the *DRL-Model* and 1.75 cm for the *Human-Avg-Model*, respectively.

It is important to note that the humanoid actor in the simulation solely relied on shoulder and elbow joint angular data to animate the reaching movements without incorporating compensatory movements such as trunk and shoulder displacements. In real scenarios, slight compensatory movements could further enhance the accuracy of the target point reaching error. These results further validate the effectiveness of our DRL-based synthetic motion data in accurately predicting the elbow joint motion during natural human movements.

### Motion Data Augmentation: Cross-Subject Evaluation

To assess the impact of integrating DRL-Data with Real-Data on the performance of our predictive models, we developed CNN-LSTM models, namely the *Human-Sparse-Model* and *Hybrid-Model* (see Section 2.5), representing a scenario with limited training data availability. Subsequently, we conducted a cross-subject evaluation by utilizing the sparse model of one subject to predict the elbow joint motion of all other participating subjects. This approach accounts for the inherent inter-subject variability, providing valuable insights into the robustness and transferability of the trained predictive models.

**Figure 10:**
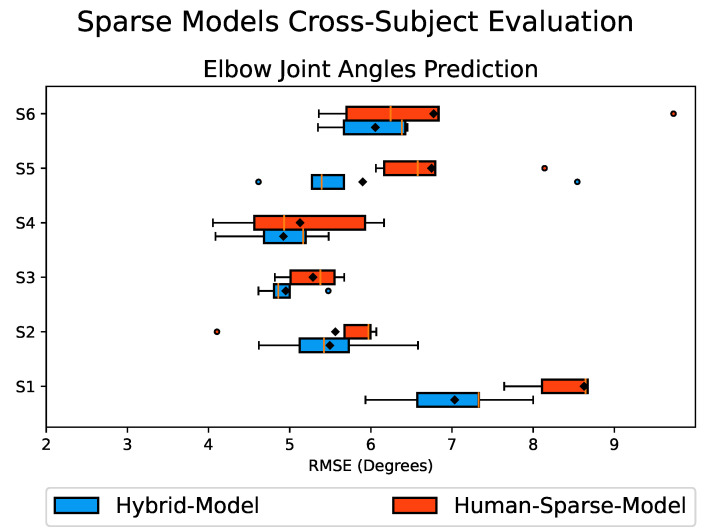
Box plot of the RMSE values comparing the predicted and actual elbow joint angles obtained from the cross-subject evaluation of the *Hybrid-Model* in blue and *Human-Sparse-Model* in red. The box size represents the range encompassing 75% of the values, with the solid vertical golden line inside indicating the median. A black diamond marker denotes the mean value. Circular markers represent outliers, and the whiskers indicate the maximum and minimum values. Smaller box sizes, along with lower mean and median RMSE values, indicate better performance.

Figure 10 presents the RMSE values obtained from the cross-subject evaluation of the *Hybrid-Model* and the *Human-Sparse-Model*, represented as a box plot. The box size indicates the range encompassing 75% of the sample values, with the solid vertical golden line inside representing the median. A black diamond marker indicates the mean value. Smaller box sizes, along with smaller mean and median values, indicate less variation in the prediction results and better overall performance.

The results depicted in Figure 10 demonstrate that the *Hybrid-Model* had improved performance for all six subjects, as indicated by its smaller box size along with the lower mean RMSE values compared to the *Human-Sparse-Model*. This can also be observed from the percentage breakdown of the performance improvement presented in Table 2. The overall cross-subject results show that the *Hybrid-Model* had an overall average RMSE value of 5.72°, whereas the *Human-Sparse-Model* had an overall average RMSE value of 6.35° with an overall average improvement of about 10% in the prediction performance. These findings highlight the potential of integrating DRL-Data with Real-Data to enhance the overall performance and robustness of the predictive model. By augmenting the subject’s data with the additional synthetic motion data, it enriches the diversity of the training dataset, contributing to improved model performance.

## 4. Discussion

We propose a DRL-based motion cloning framework for the synthetic motion generation of arm reaching movements. The synthetic motion data effectively supplement and diversify the training motion data, addressing the challenge of acquiring large motion datasets from human subjects for training a predictive model. Our evaluation results showcase the efficacy of cloned motion data in accurately predicting natural human elbow joint movements. Furthermore, motion data augmentation demonstrated the enhanced performance of the predictive model across multiple subjects in the case of the decoder based on human experiments.

We assessed the quality of the cloned motion data (DRL-Data) generated through our DRL simulation by examining its correlation with the averaged human motion data and comparing the results to that of Real-Data obtained from all participating subjects. Pearson’s correlation coefficient analysis revealed a strong similarity between the cloned DRL-Data and the motion data from the other subjects, albeit with slight variations in correlation values due to the inherent inter-individual variability. These results highlight that the synthetic dataset generated by our DRL-based motion cloning framework can be considered as an additional subject within the experiment. Such findings provide compelling evidence supporting the effectiveness of our DRL-based motion cloning framework in successfully synthesizing human-like synergistic motion.

We used synthetic motion data to train a predictive model, the *DRL-Model*, and evaluated its performance against the *Human-Avg-Model* (used as the gold standard), which was trained using the averaged human motion data from all the participating subjects. We employed metrics such as Pearson’s correlation coefficient and RMSE values to measure the linearity and average difference between the estimated and actual joint angular values. Both predictive models exhibited a similar performance, as evidenced by comparable Pearson’s correlation coefficient values and minor differences in overall RMSE values. This indicates that the synthetic motion data successfully captured the essential reaching movement synergistic patterns, enabling the effective training of the *DRL-Model*. Notably, the *DRL-Model* was tested using human motion data as input, which uniquely differs from the synthetic motion data used for training. Nonetheless, it accurately predicted the elbow joint motion across all subjects, demonstrating the robustness of the DRL-Model in accommodating inter-subject variability.

We also conducted a visual analysis of the predicted motion using a Unity 3D simulation, where a humanoid actor animated the reaching movements toward all target points. We calculated the target reaching error to assess the accuracy of the estimations. The results demonstrated that both the *DRL-Model* and the *Human-Avg-Model* successfully reached the target points with reasonable accuracy. While there were slight variations in a few cases, overall, both models produced similar overall target reaching errors. It is important to note that the humanoid actor in the simulation solely relied on shoulder and elbow joint angular data to animate the reaching movements without incorporating compensatory movements. Therefore, the target reaching errors can be further improved in real scenarios by incorporating slight compensatory movements such as trunk and shoulder displacements.

Finally, we investigated the impact of integrating DRL-Data with the Real-Data for training purposes to enhance the performance of the predictive models. We trained predictive models with sparse training data, namely *Human-Sparse-Model* and *Hybrid-Model*, and evaluated their prediction accuracy through a cross-subject evaluation. The results reveal that the *Hybrid-Model* outperformed the *Human-Sparse-Model*, demonstrating improved performance in all six subjects, as indicated by the smaller box plot size and lower overall average RMSE values. These findings highlight the potential of integrating DRL-Data with Real-Data, leading to the enhanced overall performance and robustness of the predictive model. By augmenting the subject’s data with additional synthetic motion data, the training dataset becomes more diverse, contributing to improved model performance.

In this study, we explored synthetic motion generation with a specific focus on fundamental horizontal-plane reaching movements and its utilization in motion data augmentation to improve the predictive model’s performance. Looking ahead, our research trajectory entails an in-depth exploration of the domain of synthetic motion data generation. We are striving to enhance the accuracy and diversity of the synthetic dataset through the implementation of advanced DRL techniques to encompass a broader range of dynamic movements and scenarios.

## 5. Conclusions

This study unveils the potential of synthetically generated motion data using a DRL-based motion learning approach to accurately replicate human-like synergistic arm movements and their effectiveness in training predictive models capable of accurately predicting actual human arm movements.

We present a novel DRL-based motion cloning framework designed explicitly for synthesizing motion data for arm reaching movements. Through our analysis, we confirmed that the synthetic motion data closely resemble the characteristics of motion data obtained from human subjects and effectively capture the synergistic patterns of the arm reaching movements, enabling the training of an accurate predictive model. Our trained model demonstrates the ability to predict elbow joint motion across diverse human subjects, achieving an overall average RMSE value of 5.14° and accurately reaching the target points. Notably, our results highlight the significant advantages of integrating synthetic motion data with actual motion data from human subjects during training, enhancing the performance and robustness of the predictive models in a cross-subject evaluation setting, with an overall average RMSE value of 5.72°.

This initial investigation showcases the potential of the proposed DRL-based motion cloning framework in successfully synthesizing and leveraging synthetic motion data to enhance the accuracy and reliability of predictive models in capturing natural human-like movements. Our evaluations yield compelling evidence, affirming the ability of the cloned motion data to accurately predict natural elbow motion across multiple subjects. Moreover, the cloned motion data can not only supplement limited data availability but also diversify the training data, contributing to improved generalization. These findings have significant implications for creating comprehensive synthetic motion dataset resources for diverse arm movements and advancing strategies for automated prosthetic elbow motion.

## Figures and Tables

**Table 1:** Interpretation of Pearson correlation coefficient.

Range of *r*	Degree of Relationship
−1.0≤r≤−0.7	A strong negative linear relationship
−0.7≤r≤−0.3	A distinct negative linear relationship
−0.3≤r≤−0.1	A weak negative linear relationship
−0.1≤r≤+0.1	Not a linear relationship
+0.1≤r≤+0.3	A weak positive linear relationship
+0.3≤r≤+0.7	A distinct positive linear relationship
+0.7≤r≤+1.0	A strong positive linear relationship

**Table 2:** Percentage breakdown of sparse models’ cross-subject evaluation performance.

Model	Mean RMSE Value Human-Sparse-Model	Mean RMSE Value Hybrid-Model	Percentage Improvement
S1	8.63	7.03	18.48%
S2	5.56	5.50	1.22%
S3	5.29	4.95	6.34%
S4	5.13	4.92	4.01%
S5	6.75	5.90	12.58%
S6	6.77	6.05	10.61%

## Data Availability

Not applicable.

## Abbreviations

The following abbreviations are used in this manuscript:

ADL	Activities of Daily Living
ANN	Artificial Neural Network
CNN	Convolutional Neural Network
CNN-LSTM	Convolutional Long Short-Term Memory Neural Network
DOF	Degrees of Freedom
DRL	Deep Reinforcement Learning
EMG	Electromyography
IMU	Inertial Measurement Unit
LSTM	Long Short-Term Memory
MDP	Markov Decision Processes
RBFN	Radial Basis Function Network
RMSE	Root Mean Squared Error
SAC	Soft Actor-Critic
TMR	Targeted Muscle Reinnervation
